# Supplementary material for: Digital Health Literacy of Adolescents and Its Association with Vaccination Literacy: The First Evidence from Lithuania
Source: Epidemiologia (Basel). 2025 Nov 3;6(4):73. doi: 10.3390/epidemiologia6040073 (PMC12641912; doi:10.3390/epidemiologia6040073)
Supplement: Supplementary file 1 [file epidemiologia-06-00073-s001.zip › Table S1.Descriptive results for the HLS-DIGI items.pdf]

Table S1. Descriptive results for the HLS-DIGI items

Item number	Question: "When you search online for information on health, how easy or difficult is it for you"	Mean (SD)	Percentages of "very difficult" or "difficult" responses (%)
1	to judge whether the information is reliable?	1.64 (0.48)	35.8
2	to judge whether the information is offered with commercial interest?	1.73 (0.44)	26.5
3	to understand the information?	1.90 (0.30)	10.3
4	to use the information to help solve a health problem?	1.81 (0.39)	18.5
5	to judge whether the information is applicable to you?	1.73 (0.45)	27.1
6	to find the exact information you are searching for?	1.68 (0.47)	32.1
7	to visit different websites to check whether they provide similar information about a topic?	1.91 (0.29)	9.1
8	to use the proper words or search query to find the information you are looking for?	1.88 (0.33)	12.1
