# Supplementary material for: Digital Health Literacy of Adolescents and Its Association with Vaccination Literacy: The First Evidence from Lithuania
Source: Epidemiologia (Basel). 2025 Nov 3;6(4):73. doi: 10.3390/epidemiologia6040073 (PMC12641912; doi:10.3390/epidemiologia6040073)
Supplement: Supplementary file 1 [file epidemiologia-06-00073-s001.zip › Table S2.Descriptive results for the HLS-VAC items.pdf]

Table S2. Descriptive results for the HLS-VAC items

Item number	On a scale from very easy to very difficult, how easy would you say it is …	Mean (SD)	Percentages of "very difficult" or "difficult" responses (%)
1	to find information on recommended vaccinations for you or your family?	3.04 (0.78)	19.7
2	to understand why you or your family may need vaccinations?	3.23 (0.65)	10.1
3	to judge which vaccinations you or your family may need?	3.07 (0.83)	20.3
4	to decide if you should have a flu vaccination?	3.26 (1.01)	14.6
